# Rice and millet cultivated in Ha Long Bay of Northern Vietnam 4000 years ago

**DOI:** 10.3389/fpls.2022.976138

**Published:** 2022-11-02

**Authors:** Weiwei Wang, Kim Dung Nguyen, Hai Dang Le, Chunguang Zhao, Mike T. Carson, Xiaoyan Yang, Hsiao-chun Hung

**Affiliations:** ^1^ Department of Archaeology and Natural History, The Australian National University, Canberra, ACT, Australia; ^2^ Vietnam Association of Archaeology, Hanoi, Vietnam; ^3^ Institute of Archaeology, Vietnam Academy of Social Science, Hanoi, Vietnam; ^4^ Department of Archaeology, Wuhan University, Wuhan, China; ^5^ Micronesian Area Research Center, University of Guam, Mangilao, Guam, GU, United States; ^6^ Key Laboratory of Western China’s Environmental Systems (Ministry of Education), College of Earth and Environmental Sciences, Lanzhou University, Lanzhou, China

**Keywords:** agriculture, phytolith, starch, rice, millet, Ha Long Bay, Vietnam, Southeast Asia

## Abstract

Research has generally outlined that the Neolithic East Asian farmers expanded into Southeast Asia, leading to substantial social and cultural transformations. However, the associated archaeobotanical evidence until now has been insufficient to clarify the exact timing, dispersal route, and farming package of the emergence of agriculture in Mainland Southeast Asia. To clarify these issues, the micro-plant remains of phytolith and starch from three Neolithic sites in Ha Long Bay were extracted and analyzed. This study validates the earliest evidence of co-cropping in northern Vietnam, involving the cultivation of rice together with foxtail millet at 4000 years BP or slightly earlier. Moreover, the results indicate that at least two patterns of subsistence strategy were practiced simultaneously during the initial farming phase in the region. The Trang Kenh people, a regional variant of the Phung Nguyen cultural group often have been seen as the first farmers in northern Vietnam, and they mainly practiced a cereal-based subsistence strategy with more vital cultural characteristics of southern China origin. Meanwhile, the Ha Long people, mainly composed of indigenous hunter-gatherer descendants, continued to utilize a wide range of their preferred plant resources such as taros, yams, and acorns, while they absorbed and incorporated new elements such as millet and rice into their food system. This study provides solid information to understand the diverse economic systems among different cultural groups in Vietnam.

## Introduction

Several studies based on archaeology, linguistics, and genetics have suggested that farming groups with rice and/or millet domestication originated from central China and expanded into Mainland Southeast Asia through southern China, admixing with or replacing the indigenous hunter-gatherers around 4000 years BP (Before Present) (e.g., Bellwood, 2005; Zhang and Hung, 2010; Higham et al., 2011; Lipson et al., 2018; McColl et al., 2018; Matsumura et al., 2019; Yang et al., 2020). The arrival of rice and millet agriculture and their domesticators led to significant social, cultural, and economic transformations in Mainland Southeast Asia, including technological advancement, demographic expansion, and cultural complexity.

**Figure 1:**
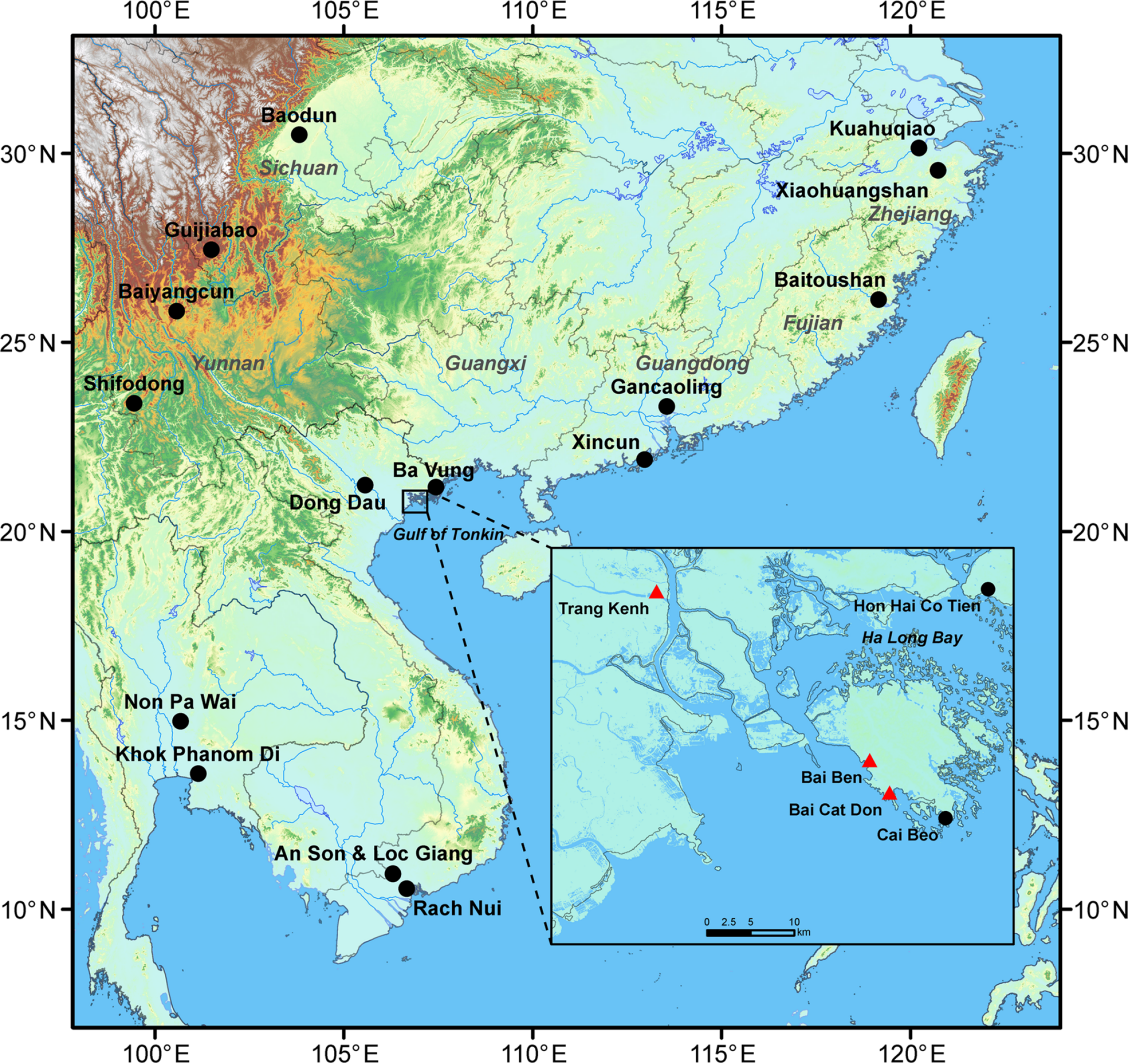
Locations of the three major studied sites (shown in red triangles) and other major sites mentioned in the text (shown in black dots).

Recent archaeobotanical studies have confirmed that both rice and millet agriculture occurred in southern China at about 5000-4800 cal. years BP. For instance, phytoliths from rice (*Oryza sativa*) and broomcorn millet (*Panicum miliaceum*) were discovered from the Tanshishan cultural layer (ca. 5000-4500 cal. years BP) at Baitoushan in coastal Fujian (Dai et al., 2021). Gancaoling in the Pearl River Delta of Guangdong documented the co-cultivation of rice and foxtail millet (*Setaria italica*) around 4800-4600 cal. years BP (Deng et al., 2022a). In the inland terrain of southwest China, rice and millet mixed cropping emerged in Guijiabao in southern Sichuan about 5000 cal. years BP (Zhao and Chen, 2011; Huan et al., 2022). The earliest crop package comprising foxtail millet, broomcorn millet, and soybean began around 4650 cal. years BP in Baiyangcun in northwestern Yunnan (Dal Martello et al., 2018; Dal Martello, 2022). Until now, the southernmost Neolithic site with evidence of rice-millet cultivation in Yunnan was from Shifodong, where rice and foxtail millet were retrieved from a context dated to ca. 3400-3100 years BP (Zhao, 2010; Dal Martello, 2022) (**Figure 1**).

In Mainland Southeast Asia, evidence related to rice comes from a few Phung Nguyen sites in northern Vietnam (Nguyen, 1998) and much farther south in the Mekong Delta region, such as at An Son and Loc Giang, dating back to 4200-3150 cal. years BP (Bellwood et al., 2011; Barron et al., 2017). Additional early rice remains were recovered from the Neolithic coastal site of Khok Phanom Di in Thailand, dated 4000-3500 cal. years BP (Thompson, 1996; Higham and Thosarat, 2005; Higham et al., 2011). So far, the earliest evidence of foxtail millet has been from Non Pa Wai in the Khao Wong Prachan Valley, where a single foxtail millet grain was dated to 4470-4200 cal. years BP, but rice was dated no earlier than 3000 BP at this same site (Weber et al., 2010). Before this study, Rach Nui (3500-3300 cal. years BP) in coastal southern Vietnam was the only known site with rice and foxtail millet, both from the same cultural layer. However, these crops were more likely acquired through trade or exchange from other farming societies (Castillo et al., 2018) (**Figure 1** and **Table 1**).

**Table 1:** Major sites with evidence of rice and millets (mark as ×) in Mainland Southeast Asia during the Neolithic period.

Country	Site	Province	Age (BP)	Collecting method	Rice	Foxtail millet	Broomcorn millet	Reference
Vietnam	Dong Dau	Vinh Phuc	3400-2800	Flotation	×			Nguyen, 2002; Nguyen, 2013; Nguyen,2017
	Thanh Den	Vinh Phuc	3700-2600	Flotation	×			Nguyen, 2017
	Xuan Kieu	Hanoi	3500-3200	Impression	×			Nguyen, 1998; Nguyen, 2017
	Tu Son	Bac Ninh	3500-3200	Impression	×			Nguyen, 1998
	Bai Cu	Thanh Hoa	4000-3500	Impression	×			Nguyen, 1998
	Bai Man	Thanh Hoa	3500-3200	Impression	×			Nguyen, 1998
	Thach Lac	Ha Tinh	4000-3500	Pollen	×			Nguyen, 2013
	Rach Nui	Long An	3500-3300	Flotation, phytolith	×	×		Castillo et al., 2018; Weisskopf, 2018
	An Son	Long An	4200-3150	Impression, microCT	×			Bellwood et al., 2011; Barron et al., 2017
	Loc Giang	Long An	4000-3300	Impression, microCT	×			Barron et al., 2017
Cambodia	Krek 52/62	Kompong Cham	4620-3690	Impression	×			Albrecht et al., 2000; Vanna, 2002
	Samrong Sen	Kampong Chhnang	3800-3200	Impression	×			Vanna, 2002
	Mlu Prei	Preah Vihear	3500-1300	Impression	×			Vanna, 2002
Thailand	Banyan Valley Cave	Mae Hong Son	5500-1300	Dry-sieving	×			Yen, 1977
	Ban Chiang	Udon Thani	3600-3100	Flotation, dry-sieving, pollen, impression	×			Higham, 1989; Kealhofer and Penny, 1998; Higham et al., 2015
	Non Nok Tha	Khon Kaen	3500-3000	Impression	×			Bayard, 1972; Vincent, 2002; Higham et al., 2015
	Khok Charoen	Lopburi	3500-3150	Impression	×			Vincent, 2002
	Non Pa Wai	Lopburi	4470-2700	Flotation	× (after 3000 BP)	×	×	Pigott et al., 2006; Weber et al., 2010
	Non Mak La	Lopburi	4100-2700	Flotation, phytolith	× (after 3000 BP)	×	×	Pigott et al., 2006; Weber et al., 2010
	Nil Kham Haeng	Lopburi	3350-2500	Flotation, phytolith	× (after 3000 BP)	×	×	Pigott et al., 2006; Weber et al., 2010
	Tha Kae	Lopburi	3700-3100	Phytolith, impression	×			Kealhofer, 1998; Rispoli et al., 2013; Higham, 2014
	Ban Lum Khao	Nakhon Ratchasima	3400-2600	Flotation, impression	×			Tayles et al., 2000; Higham and Thosarat, 2004; Higham et al., 2015
	Khok Phanom Di	Chonburi	4000-3500	Impression, dry-sieving, hand-picked	×			Thompson, 1996; Higham and Thosarat, 2012; Higham, 2014

Overall, uncertainty has surrounded whether rice and millet domesticates had appeared in Mainland Southeast Asia simultaneously in the first place. The archaeobotanical work has been insufficient to reconstruct the initial dispersal of the timing, route(s), and the combination of crops for early farming into Mainland Southeast Asia. Presumably, the spread of Neolithic farmers and their farming cultures from southern China to Mainland Southeast Asia occurred rapidly and through both inland river systems and coastal roads (Higham, 2017; Higham, 2019; Guedes et al., 2020; Deng et al., 2022a; Deng et al., 2022b). The northeastern regions of Mainland Southeast Asia, neighboring both inland and coastal southern China, must have been the first accommodation for the early farmers from the north. Therefore, the Red River floodplain and coastal areas around the Gulf of Tonkin are crucial for understanding the dispersal of early rice and millet and their domesticators into Mainland Southeast Asia.

In northern Vietnam, despite some sporadic reports of pottery impressions from rice husks, or a few carbonized rice (Nguyen, 1998; Nguyen, 2013; Nguyen, 2017), the systematic archaeobotanical work to authenticate the evidence of agriculture in this region has been somewhat limited. In this regard, two of the most representative Neolithic groups, Trang Kenh and Ha Long, both coexisted around 4000 cal. years BP in the northeastern coast of Vietnam, and therefore they are ideal for investigating the emergence of rice and millet cultivation in Mainland Southeast Asia. This study investigates micro-plant remains (phytolith and starch) extracted from stone tools excavated from three sites belonging to these two cultural groups in northeastern Vietnam. The new data obtained from this study are incorporated with previously collected data from the Cai Beo site on Cat Bat Island (Wang et al., 2022). All four sites discussed in this study were excavated when the floatation method for collecting macro-plant remains was not yet applied in Vietnam.

## Site description

**Figure 2:**
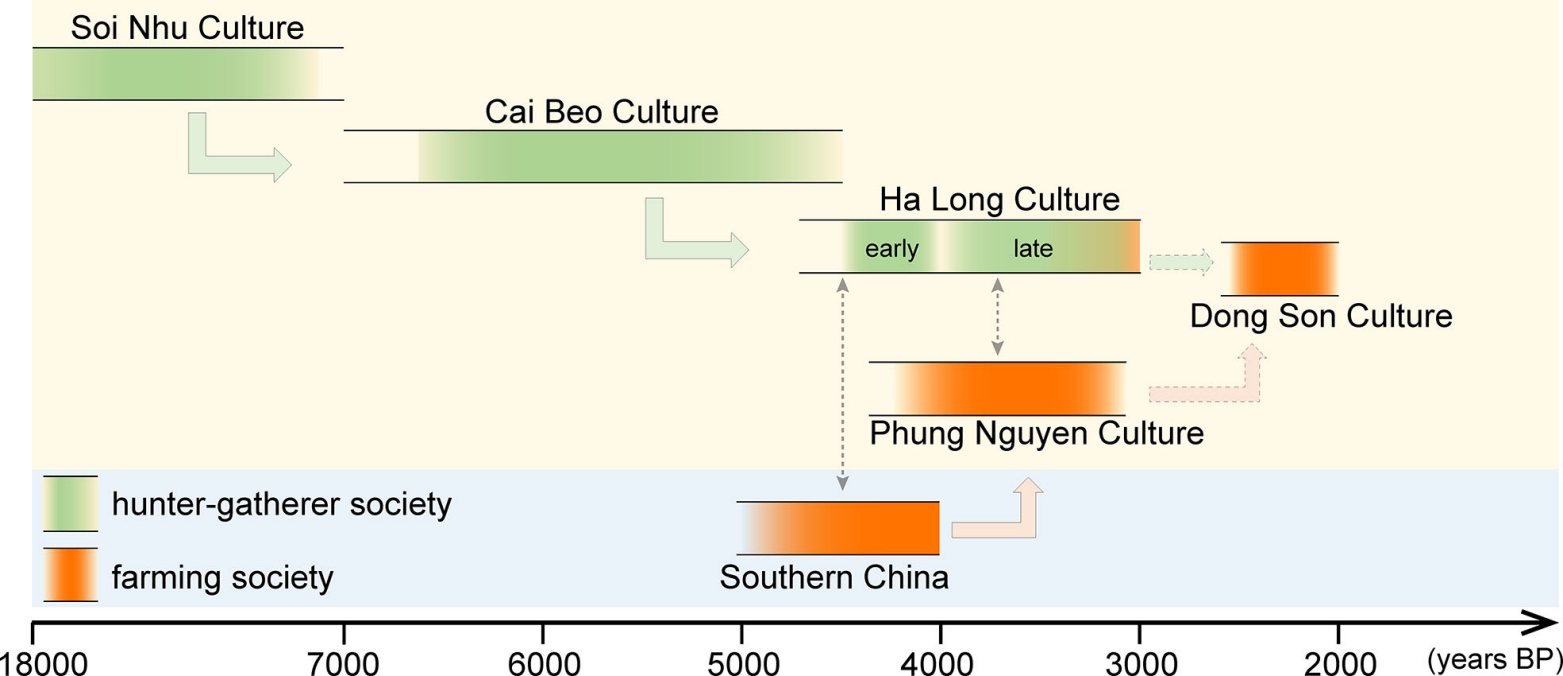
Archaeological chronology and cultural sequence in Ha Long Bay, noting relations with southern China (interactions between two cultural groups: shown in black dotted-line arrows).

About 4000 years ago, two cultural groups, Ha Long and Trang Kenh, coexisted around the Gulf of Tonkin. The Ha Long group (ca. 4500-3000 cal. years BP) was a developed maritime-oriented society, supported mainly by fishing and hunting, augmented by perhaps a small amount of farming (Nguyen, 2019). Many archaeologists have suggested that a deep root of the Ha Long group can be traced back to the earlier Cai Beo group in the same region, which had developed from the Late Pleistocene Hoabinhian-Bacsonian tradition (Nguyen et al., 2004; Nguyen, 2009). Overlaping in time with the Ha Long group, the Trang Kenh group (ca. 4000-3200 cal. years BP) contains strong characteristics of the Neolithic Phung Nguyen (ca. 4100-3200 cal. years BP), often regarded as the most thriving early farming society in northern Vietnam that likely could be traced back to a southern China origin (**Figure 2**).

While the Trang Kenh group concentrated in the Red River Delta in Hai Phong Municipality, the Ha Long group ranged through the coastal areas and nearby islands of the Ha Long Bay. These two groups, defined by the Vietnamese archaeologists, show differences in many ways, such as their artifact assemblages, burial practices, and other cultural aspects. For example, most of the human burials of the Ha Long group are in the flexed position, the typical practice of hunter-gatherers in Vietnam since the Hoabinhian cultural phase more than 10,000 years ago (Higham, 2013; Hung et al., 2017; Matsumura et al., 2019). Some Ha Long burials contain goods such as typical shouldered axes/adzes, pebble pointed tools, grinding stone "Ha Long Mark", bracelets with D/rectangular section and pottery. In contrast, the burial position of the Trang Kenh group is extended, like the cemeteries in early farming societies in southern China and Phung Nguyen sites. Moreover, these burials often contain grave goods such as pottery vessels, lithic tools, and shell or jade ornaments.

To collect the ancient micro-plant remains, this study analyses 20 stone tools excavated from two Ha Long cultural sites, Bai Ben and Bai Cat Don, and the representative Trang Kenh cultural group site, the Trang Kenh site itself.

### Bai Ben site

Bai Ben site (N20°46’44”, E106°58’20”) is located in a present-day fishing port surrounded by limestone mountains on the west side of Cat Ba Island (Nguyen and Clarkson, 2013) (**Figure 1**). The site covers an area of 5000 m^2^ distributed along the main road of the island and the western seashore. An area of 142.5 m^2^ was excavated over two seasons in 1999 and 2001 (Pham, 2003). Excavations at Bai Ben consistently have uncovered a single cultural layer of about 80 cm in thickness. In addition, the site has yielded tens of thousands of drill points, cores, and flakes, suggesting it was a workshop specializing in drill point manufacture (Nguyen and Clarkson, 2013).

**Figure 3:**
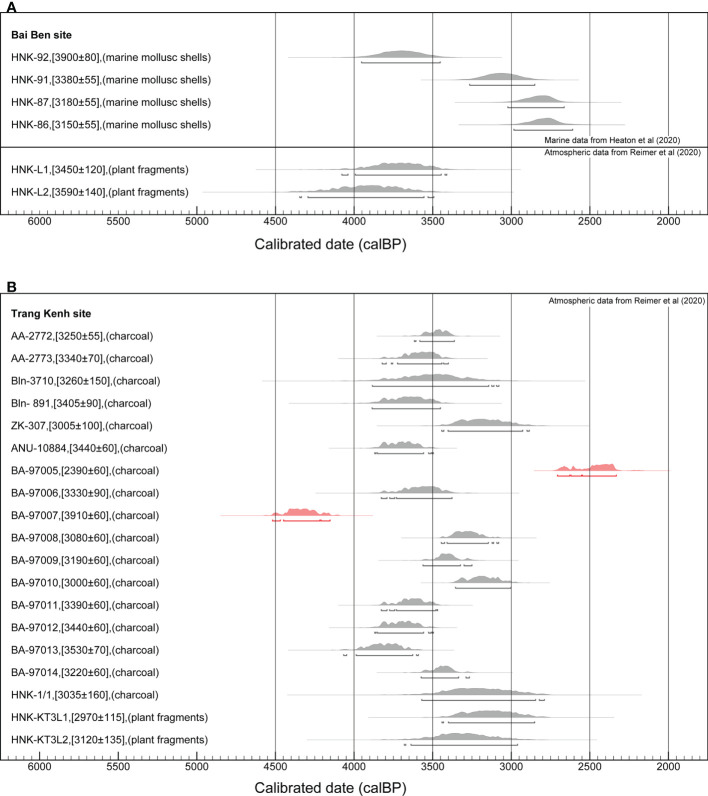
Radiocarbon dates form Bai Ben **(A)** and Trang Kenh **(B)**. All dates were calibrated with OxCal v4.4.2 (Ramsey, 2009) and presented at 2σ probability, using details as reported in **Table 2**.

Six ^14^C dates obtained from mollusc shells and plant fragments in Bai Ben suggest an occupation at 4100-3100 cal. years BP (Nguyen and Tran, 2009; Nguyen and Clarkson, 2013) (**Figure 3** and **Table 2**). Bai Ben has yielded ground shouldered and stepped or rectangular axes/adzes, sandstone saw blades, chert ground/flaked pointed tools, grinding stones, stone hammers, anvils, a stone spearhead, and a small number of ornaments including slate and nephrite beads and bracelet fragments (Nguyen and Clarkson, 2013). In addition, earthenware vessels and fragments with rectangular everted rims, curved rims with outer roofs, stamped, incised with “S” shape, and short parallel line motifs, present the typical pottery characteristics of the Ha Long group.

**Table 2:** Radiocarbon dates from Bai Ben and Trang Kenh.

Site name	Sample No.	14C dates BP	Material	Cal. BP (2σ)	Context	Reference
Bai Ben	HNK-92	3900 ± 80	marine mollusc shells (species unknown)	3950 - 3453	Layer 4	Nguyen and Tran, 2009; Nguyen and Clarkson, 2013
	HNK-91	3380 ± 55	marine mollusc shells	3262 - 2852	Sterile bottom	Nguyen and Tran, 2009; Nguyen and Clarkson, 2013
	HNK-87	3180 ± 55	marine mollusc shells	3021 - 2664	Sterile bottom	Nguyen and Tran, 2009; Nguyen and Clarkson, 2013
	HNK-86	3150 ± 55	marine mollusc shells	2981 - 2611	Layer 4	Nguyen and Tran, 2009; Nguyen and Clarkson, 2013
	HNK- L1	3450 ± 120	plant fragments	4078-3411	plant fragments in pottery	Nguyen and Tran, 2009
	HNK- L2	3590 ± 140	plant fragments	4346-3493	plant fragments in pottery	Nguyen and Tran, 2009
Trang Kenh	AA-2772	3250 ± 55	charcoal	3616-3362	160-180 cm, M1 Trench 1, 1986 excavation	Nguyen, 1996
	AA-2773	3340 ± 70	charcoal	3821-3400	180-200 cm, M2 Trench 2, 1986 excavation	Nguyen, 1996
	Bln-3710	3260 ± 150	charcoal	3884-3079	160-180 cm, M1 Trench 1, 1986 excavation	Nguyen, 1996
	Bln- 891	3405 ± 90	charcoal	3885-3451	190 to 210 cm, Trench 1A, 1969 excavation	Nguyen, 1996
	ZK-307	3005 ± 100	charcoal	3442-2885	140 cm, Trench 1B, 1969 excavation	Nguyen, 1996
	ANU-10884	3440 ± 60	charcoal	3868-3495	Layer 5, 175 to 190 cm	Tang and Nguyen, 2003
	BA-97005	2390 ± 60	charcoal	2706-2332	layer L2	Nguyen, 2005a
	BA-97006	3330 ± 90	charcoal	3827-3377	layer L2	Nguyen, 2005a
	BA-97007	3910 ± 60	charcoal	4519-4153	layer L2	Nguyen, 2005a
	BA-97008	3080 ± 60	charcoal	3445-3080	layer L3	Nguyen, 2005a
	BA-97009	3190 ± 60	charcoal	3561-3250	layer L3	Nguyen, 2005a
	BA-97010	3000 ± 60	charcoal	3354-3002	layer L3	Nguyen, 2005a
	BA-97011	3390 ± 60	charcoal	3827-3469	layer L4	Nguyen, 2005a
	BA-97012	3440 ± 60	charcoal	3868-3495	layer L4	Nguyen, 2005a
	BA-97013	3530 ± 70	charcoal	4066-3591	layer L5	Nguyen, 2005a
	BA-97014	3220 ± 60	charcoal	3573-3267	layer L5	Nguyen, 2005a
	HNK-1/1	3035 ± 160	charcoal	3570-2789	140 to 160 cm	Nguyen, 2005a
	HNK-KT3L1	2970 ± 115	plant fragments	3441-2851	plant fragments in pottery	Nguyen, 2005a
	HNK-KT3L2	3120 ± 135	plant fragments	3680-2960	plant fragments in pottery	Nguyen, 2005a

Calibrated through OxCal v4.4.2 (Ramsey, 2009) to 2σ cal. BP. Charcoal and plant fragment were calibrated with the INTCAL20 dataset (Reimer et al., 2020), and marine shells were calibrated with the standard MARINE20 dataset (Heaton et al., 2020) without a local marine reservoir (ΔR) calculation.

**Figure 4:**
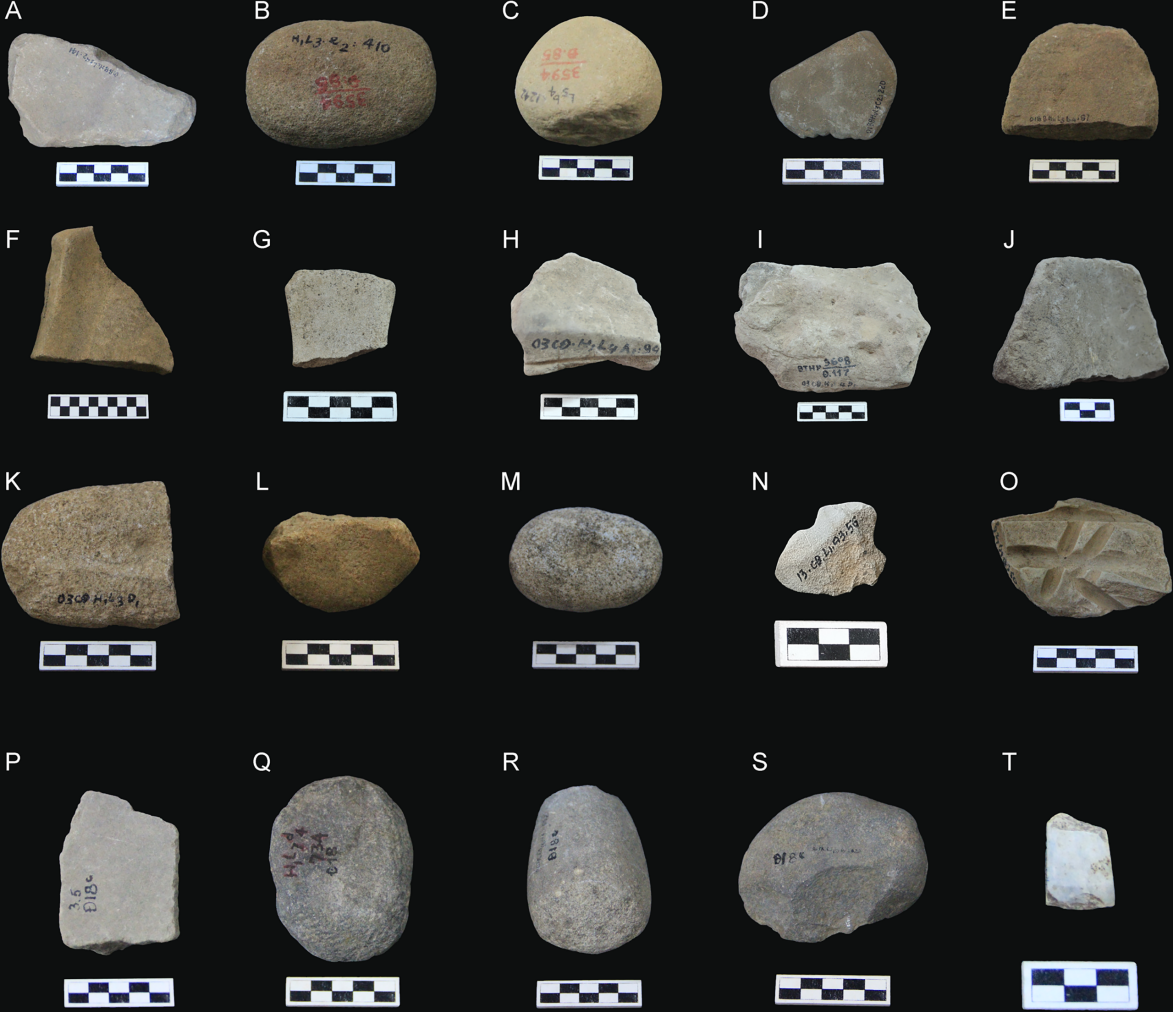
Stone tools from Bai Ben **(A–E)**, Bai Cat Don **(F–O)**, and Trang Kenh **(P–T)** for this study; **(A, F–K, P)** Grinding stone, **(B, C, L, Q, R)** Muller/Pounder, **(D, O)** Grinding stone tools “Ha Long Mark”, **(E)** Short axe, **(M)** Pitted pebble, **(N)** Shouldered axe fragment, **(S)** Chopper, **(T)** Adze.

Vietnamese archaeologists generally regard Bai Ben as a late Ha Long cultural site. However, much evidence from the site has reflected the cultural interactions between Ha Long and other groups, such as Phung Nguyen in the Red (Hong), Da, Lo, and Day River, Hoa Loc in northwestern Thanh Hoa Province, and some contemporary workshop sites like Trang Kenh in Hai Phong (Nguyen, 2001; Pham, 2003). Five stone tools from Bai Ben are analyzed during this study (**Figures 4A–E** and **Table 3**).

**Table 3:** Identification of starch grains recovered from the studied sites.

Tool	Field number	Tool type	Starch Type										Total
			Aroid		Yam		Palm	Ginger	Millet	Acorn	Bean	Rice	
			Ia	Ib	IIa	IIb	III	IV	V	VI	VII	VIII	
**Bai Ben site**													
1	01 BB H11 L3 a2: 141	Grinding stone	5		1		1		3				10
2	BB 99. TS2. T1 M1 L3. e2: 410	Muller/Pounder	11		6		1						18
3	BB 99. TS2. T1 L5 b4: 1242	Muller/Pounder	6		2		1	1					10
4	01 BB H11 L3 c2: 220	Grinding stone tool “Ha Long Mark”	1						1				2
5	01 BB H11 L3 b4. 87	Short axe	6		2	3							11
Total			29		11	3	3	1	4				51
Total %			56.86%		21.57%	5.88%	5.88%	1.96%	7.84%				100.00%
**Bai Cat Don site**													
1	13 CD H1 L1 b3. 32	Grinding stone	1						1				2
2	13 CD H1 L1 b5	Grinding stone	6						3				9
3	03 CD H1 L4 A1: 94	Grinding stone	9	2	3				1	1			16
4	03 CD H1 L4 D5	Grinding stone	3				1						4
5	03 CD H1 L3 D2. 76	Grinding stone			1		1		2				4
6	03 CD H1 L3 D1	Grinding stone	1		2		2			1			6
7	13 CD H1 L1 a5.8	Muller/Pounder	1										1
8	03 CD H1 L4 A4	Pitted pebble			1	1			2				4
9	13 CD L1 a3. 56	Shouldered axe fragment							1		2		3
10	03 CD H1 L2 b2	Grinding stone tool “Ha Long Mark”	11						1				12
Total			32	2	7	1	4		11	2	2		61
Total %			52.46%	3.28%	11.48%	1.64%	6.56%		18.03%	3.28%	3.28%		100.00%
**Trang Kenh site**													
1	35 D18 C	Grinding stone	6						4			5	15
2	H1 L7 d4 734 D18	Muller/Pounder	5					1	10			2	18
3	96 TK L3 (5) D4 552	Muller/Pounder							2				2
4	96 TK L5 (5) D622	Chopper	2										2
5	86 TK H1 L5 60	Adze	1										1
Total			14					1	16			7	38
Total %			36.84%					2.63%	42.11%			18.42%	100.00%

### Bai Cat Don site

Bai Cat Don site (N20°44’43.40”, E106°59’33.74”) is a residential site located in the southwestern corner of Cat Ba Island in Ha Long Bay, about 4 km from Bai Ben (Bui, 2016) (**Figure 1**). Two excavations in 2002 and 2013 uncovered a total 78 m^2^ (Nguyen et al., 2005; Bui, 2016). The cultural layer of Bai Cat Don is about 80-100 cm. Like Bai Ben, Bai Cat Don is regarded as a late Ha Long site. The typical characteristics of the stone artifacts are grinding stones with multiple grooves often named as the “Ha Long Mark”, small shouldered axe/adzes, and pebble pointed tools. The pottery artifacts are broken pieces of containers and cooking utensils such as pots, low-bottomed bowls, standard bowls, and others. Similar to Bai Ben pottery, they are spongy because of shell fragments that were mixed into the clay matrix. In addition, some potsherds with reddish-brown color, decorated with incised curves combined with seashell imprinted edges, are typical in Hoa Loc cultural sites from Thanh Hoa Province (Bui, 2016).

So far, no radiocarbon dates are available from the Bai Cat Don site. During this study, we intended to carbon-14 date samples from Bai Ben and Bai Cat Don, but no dateable materials were available in the curation storage. Still, the similar stone tools and ceramic wares in both the manufacturing materials and stylistic output indicate a context equivalent with Bai Ben and belonging to the late Ha Long cultural phase (Bui, 2016).

This archaeobotanical study includes ten stone tools collected from 2003 and 2013 excavations at Bai Cat Don. They are six grinding stones, one muller/pounder, one pitted stone, a one-shouldered axe, and one “Ha Long Mark” artifact (**Figures 4F–O** and **Table 3**).

### Trang Kenh site

Trang Kenh site (N20°57’10”, E106°45’10’’) is located near the estuary of the Bach Dang River in Hai Phong City (**Figure 1**). It was discovered in 1968 and excavated five times, opening a total of 380.5 m^2^ (Nguyen, 2005a). The cultural layer of the site is between 1.8 m and 2.1 m thick, with abundant stone artifacts, pottery, mollusk shells, animal bones, and teeth. More than 19 ^14^C dates were obtained from the Trang Kenh site (Nguyen, 1996; Tang and Nguyen, 2003; Nguyen, 2005a), and they produce results generally in the range of 4000-3000 cal. years BP (**Figure 3** and **Table 2**).

**Figure 5:**
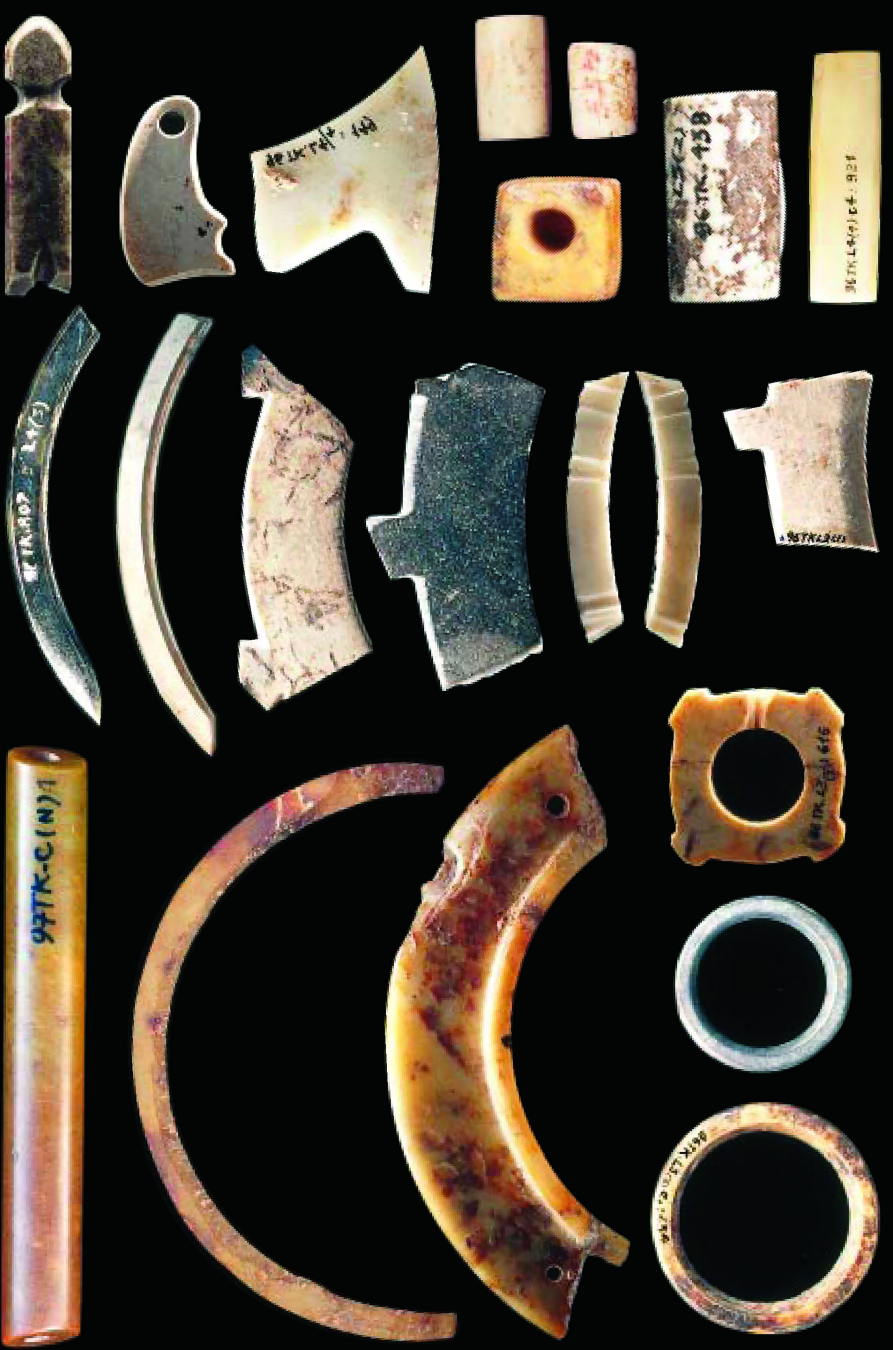
Jade ornaments excavated from the Trang Kenh jade workshop.

Many nephrite gouges and adzes were found at Trang Kenh. The most remarkable findings here were the thousands of pieces of debris and debitage from the manufacture of nephrite (jade) ornaments such as bracelets, small rings, and beads, and the associated crafting tools such as saws, chisels, and jasper drills (**Figure 5**). Trang Kenh is the largest nephrite workshop site known so far in Southeast Asia. It was a specialized manufacturing settlement for making ornaments for exchange with other communities of the Phung Nguyen and Dong Dau groups in the Red River Valley.

Burial features frequently contained finished jade ornaments (Nguyen, 1996). Three burials were reported in the 1969-1970 excavation, where one deceased was in an extended, supine position with the head pointing to the north, overlaying a pavement of pebbles in the bottom, and one grave included an offering of a trapezoidal adze (Hoang et al., 2005). In addition, many small pits were discovered on habitation living surfaces, and some showed traces of probable post holes related to the ancient house structures (Tang and Nguyen, 2003). Five stone tools from Trang Kenh were included in this study (**Figures 4P–T** and **Table 3**).

## Materials and methods: Micro-plant extraction and identification

All the stone tools analyzed in this study had been excavated by Kim Dung Nguyen and colleagues, and the objects have been stored in the Hai Phong Museum and Institute of Archaeology (VASS), Hanoi. As the major goal was to understand their staple food, grinding stone tools were preferred for this study, but other types of lithic tools were tested to investigate their functions. Sediments adhering to the stone tools and dust from the storerooms were collected as comparative samples to exclude the possibility of modern contamination.

The sediments and dust on each tool’s surface first were rinsed with distilled water and then cleaned in an ultrasonic bath with distilled water for five minutes to recover the residues. Next, the ultrasound mixtures were transferred into test tubes and processed to recover the micro plant remains, including starches and phytoliths, in the laboratory at the Department of Archaeology and Natural History, the Australian National University.

The extraction process of starches and phytoliths followed the procedures of previous studies (Lu et al., 2002; Yang et al., 2013; Pearsall, 2016; Deng et al., 2017; Wang et al., 2022). First, solutions of 6% H_2_O_2_ and 10% HCl were used separately to remove the organic matter and carbonate. Second, the starch grains were isolated with LST (lithium heteropolytungstate) heavy liquid (density 1.9 g/cm^3^), then mounted on a slide in a solution of 10% glycerine and 90% distilled water, and then next sealed with nail polish. Third, the residues were rinsed three times with distilled water, and then the phytoliths were separated with heavy liquid (density 2.35 g/cm^3^). After these steps, the samples were rinsed twice with distilled water and then once more with 30% ethyl alcohol. At this point, the phytoliths were mounted on the slide with Canada Balsam. All observed starch grains and phytoliths on each slide (100%) were counted, measured, and recorded under the optical microscope (Machine model: Olympus BX-51) at 400× magnification.

The identification of ancient starches was based on the modern reference collections from Vietnam (collected by the authors of this study), the database (http://cmsgd.igsnrr.ac.cn/) built by the Institute of Geographic Sciences and Natural Resources Research (IGSNRR) that contains more than 200 species of plants, particularly the rich morphological data of cereal crops and their wild relatives (Yang et al., 2018), and other related published studies from tropical and subtropical areas of Asia and the Pacific (Fullagar et al., 2006; Lentfer, 2009; Yang et al., 2013; Yao, 2016; Li, 2020). However, the observed phytoliths were very few and could not be classified taxonomically into any species. No starch was recovered from comparative samples that were collected for excluding possible modern contamination.

## Results

### Bai Ben site

**Figure 6:**
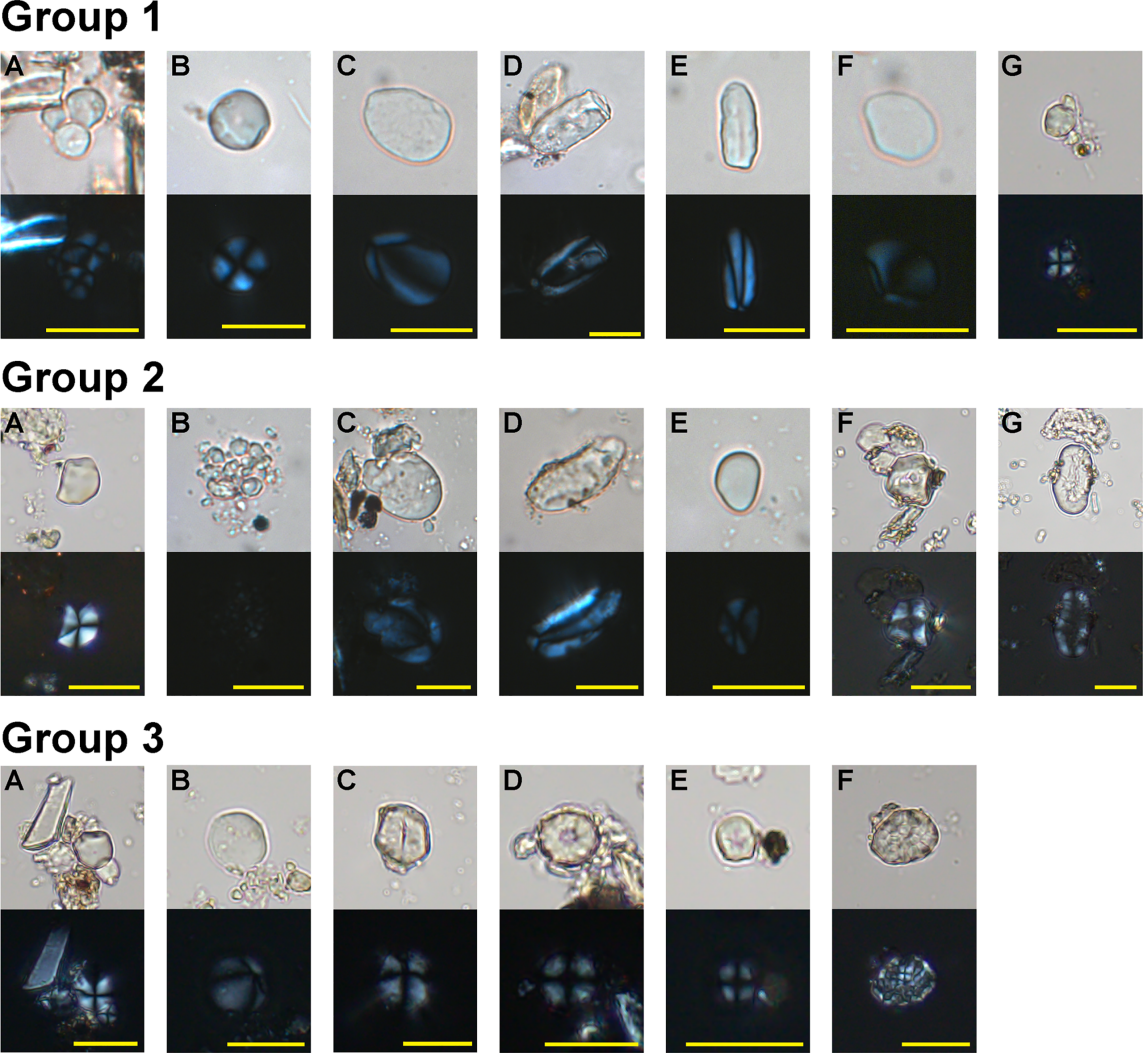
Ancient starches recovered from residues on the stone tools (under polarized and brightfield light). Group 1 from Bai Ben site: **(A, B)** Type Ia, *Colocasia* spp./*Alocasia* spp., **(C)** Type IIa, *Dioscorea alata*, **(D)** Type IIb, *Dioscorea* spp., **(E)** Type III, *Arenga* sp., **(F)** Type IV, *Zingiber* sp., **(G)** Type V, *Setaria italica*,; Group 2 from Bai Cat Don site: **(A)** Type Ia, *Colocasia* spp./*Alocasia* spp., **(B)** Type Ib, *Colocasia esculenta*, **(C)** Type IIa, *Dioscorea alata*, **(D)** Type III, *Arenga* sp., **(E)** Type V, *Setaria italica*, **(F)** Type VI, *Quercus* sp., **(G)** Type VII, *Vigna* sp.; Group 3 from Trang Kenh site: **(A)** Type Ia, *Colocasia* spp./*Alocasia* spp., **(B)** Type IV, *Zingiber* sp., **(C–E)** Type V, *Setaria italica*, **(F)** Type VIII, *Oryza sativa*. Scale bar = 20μm.

A total of 56 starch grains were recovered from the Bai Ben lithic tools (N=5), of which five starch grains could not be classified due to the lack of identifiable features. More than half of the other 51 starch grains were identified as edible Aroids (**Table 3**). They usually were round or sub-round in shape with multiple flat facets and centric hilum (**Figures 6, Group 1A, B** **;** **7A, B**) (**Table 4**). The facet starch grains appear widely in many plants with underground organs, so they are difficult to identify into species, especially when the number of recovered starches is small. However, integrated with the former large findings of these types of starches from the Cai Beo site (Wang et al., 2022), we can conclude that the inhabitants in Bai Ben inherited the tradition of extensive utilization of edible Aroids (*Colocasia* spp.; *Alocasia* spp.).

**Figure 7:**
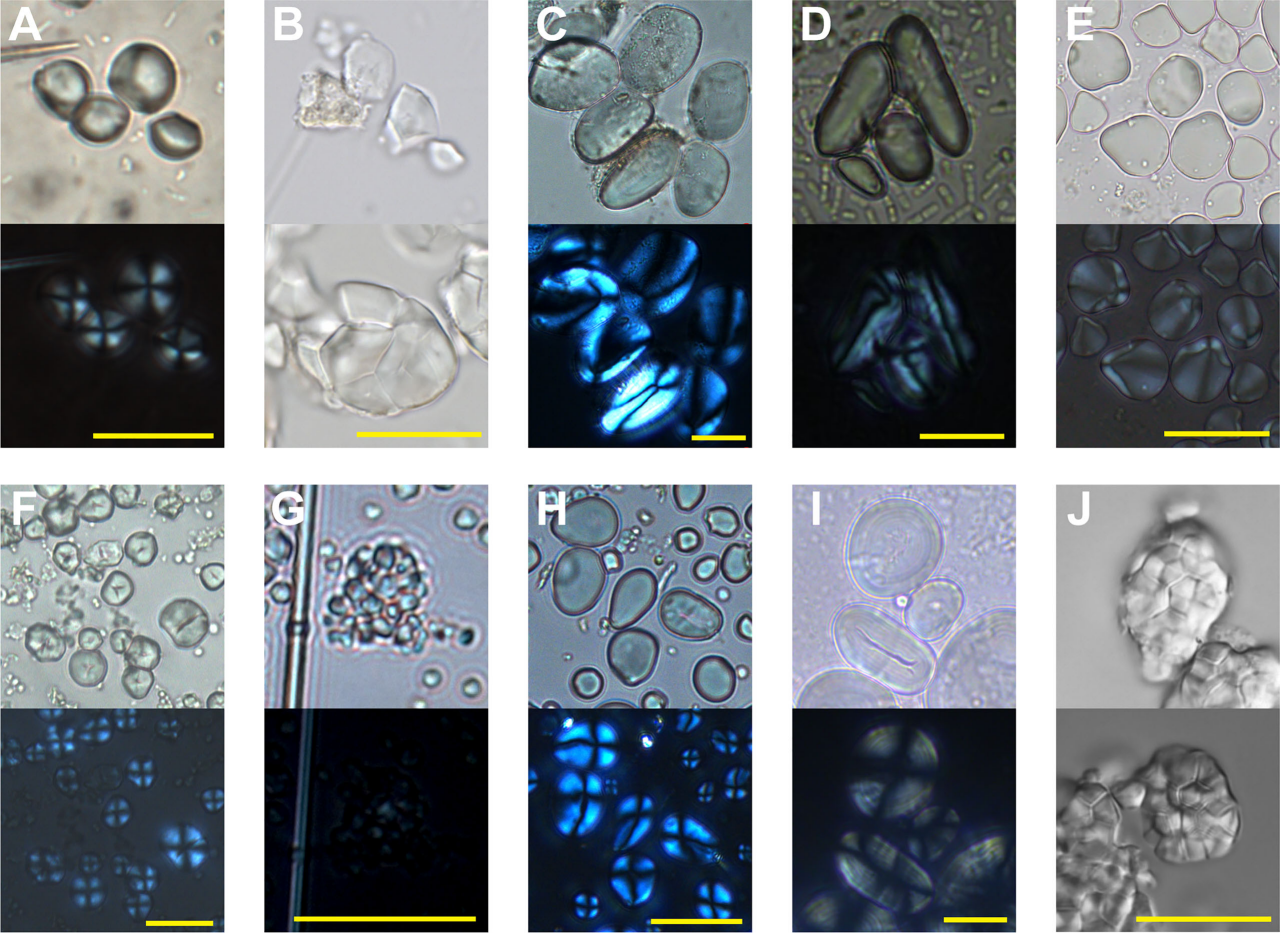
Modern starch references relevant to this study (under polarized and brightfield light). **(A)** *Colocasia konishii*, **(B)** *Alocasia macrorrhizos*, **(C)** *Dioscorea esculenta*, **(D)** *Arenga pinnata*, **(E)** *Zingiber officinale*, **(F)** *Setaria italica*, **(G)** *Colocasia esculenta*, **(H)** *Quercus francheti*, **(I)** *Vigna umbellata*, **(J)** *Oryza sativa* subsp. *japonica*. Scale bar = 20μm.

**Table 4:** Morphology of starch grains from the studied sites.

Type	Subtype	Granule shape	Size range (μm)	Mean size (μm)	Hilum	Fissures	Lamellae	Extinction cross	ID
I	Ia	Spherical/Sub-rounded/Rounded polygonal	5.64-20.42	12.69 ± 3.4	Centric	Absent	Absent	Straight	*Colocasia* spp./Alocasia spp.
	Ib	polygonal/round	< 5	< 5	Centric	Absent	Absent	Straight	*Colocasia esculenta*
II	IIa	Triangular/Elliptical	15.82-40.17	27.53 ± 7.71	Highly eccentric	Rare	Visible on large grains	Bent arms	*Dioscorea alata*
	IIb	Ovoid/Elongated ovoid	12.05-33.06	21.41 ± 6.51	Highly eccentric	Absent	Absent	Bent arms	*Dioscorea* spp.
III		Elongated ovoid	12.33-35.83	23.77 ± 7.24	Extremely eccentric	Absent	Absent	Bent arms	*Arenga* sp.
IV		Ovoid	13.3-16.26	14.78 ± 1.48	Extremely eccentric	Absent	Absent	Bent arms	*Zingiber* sp.
V		Polygonal	7.44-18.12	11.75 ± 2.92	Centric	Lined fissures	Absent	Straight	*Setaria italica*
VI		Irregular ovoid	11.16-12.95	12.51 ± 0.9	Slightly eccentric	Absent	Absent	Bent arms	*Quercus* sp.
VII		Kidney	29.68-34.66	32.17 ± 3.49	Centric	Small fissures	Absent	Bent arms	*Vigna* sp.
VIII		Polygonal	4-6.67	5.75 ± 0.83	Centric	Absent	Absent	Straight	*Oryza sativa*

Fourteen starch grains with oval or long oval shapes, highly eccentric hilum, and curved cross-arms shared similar morphological features with *Dioscorea*. Apart from three starch grains lacking the relative references to identify them into species, the other 11 starch grains, ranging from 12.05 to 38.48 μm in size, best matched with the features of the greater yam (*Dioscorea alata*) (**Figures 6, Group 1C, D**; **7C**). Three starch grains from the palm (Arecaceae) were recovered; they were narrow, elongated ovoid in shape with extreme eccentric hilum, resembling those from *Arenga* sp. (**Figures 6, Group 1E**; **7D**). One starch grain, 10.3 μm in length and 13.3 μm in width, ovoid in shape, was from ginger (*Zingiber* sp.) (**Figures 6, Group 1F**; **7E**).

Four starch grains with polygonal shapes exhibited characteristics of millets; they were classified into foxtail millets for more comparable morphological features (**Figures 6, Group 1G**; **7F**).

### Bai Cat Don site

Among the 74 starch grains recovered from the stone tools (N=10) of Bai Cat Don, 61 starches could be categorized into six groups, and the remaining 13 starch grains were unidentified. The starches from Aroids accounted for 56% of the total findings (**Table 3**). This result is consistent with our recent study from the Cai Beo site, where the edible Aroids accounted for half of the total findings (Wang et al., 2022). Thirty-two faceted round or sub-ground starch grains were from corms of *Colocasia* or *Alocasia* (**Figures 6, Group 2A**; **7A, B**).

Compared to our modern references, two clusters of compound grains with the most lengths smaller than 5 μm and faint cross arms under polarized light were identified as taro (*Colocasia esculenta*) (**Figures 6, Group 2B**; **7G**). Eight ovoid starch grains with eccentric hilum were from *Dioscorea*, among which six were the best match with *Dioscorea alata* (**Figures 6, Group 2C**; **7C**). Four starch grains with elongated ovoid shapes were identified as *Arenga* sp. (**Figures 6, Group 2D**; **7D**).

Starch grains from acorns were rare, represented in merely two granules, 12.95 μm and 11.16 μm in size, and they shared the typical physical features with *Quercus* sp. (**Figures 6, Group 2E**; **7H**).

Eleven starch grains with polygonal or spherical shape and centric hilum, ranging from 9.17 to 18.12 μm in size, sometimes with linear fissures, exhibited the distinctive features of foxtail millet (*Setaria italica*) (**Figures 6, Group 2F**; **7F**).

Two starch grains, 29.68 μm and 34.66 μm in length with an ovoid shape and small linear fissures in the hilum part, came from *Vigna* sp. (**Figures 6, Group 2G** **;** **7I**), found on the shouldered axe in Bai Cat Don.

### Trang Kenh site

In the Trang Kenh site, 38 starch grains were recovered from lithic tools (N=5), among which the cereal crops seemed quite crucial as more than half of the total starch grains were identified as millets and rice (**Table 3**). On the other hand, except for 14 starch grains from edible Aroids (*Colocasia* spp.; *Alocasia* spp.) (**Figures 6, Group 3A**; **7A, B**), no starch was found from yams, acorns, or palms at Trang Kenh, reflecting a different plant consumption mode apart from the other two sites mentioned above.

One starch grain from Ginger (*Zingiber* sp.) is 16.26 μm in size with a nearly round shape, extremely eccentric, and protruding hilum (**Figures 6, Group 3B**; **7E**).

Sixteen starch grains with polygonal shapes fall into sizes between 9.09 μm and 16.52 μm; their morphologies were most comparable with foxtail millet (*S. italica*) after being compared with modern millets and their wild relatives (**Figures 6, Group 3C**; **7F**) (Yang et al., 2012). Some parts of them showed damaged features after grinding, such as rough surface, hollowed hilum, widened and weakened extinction cross (**Figures 6, Group 3D, E**).

Notably, two groups of compound starch grains that shared the typical features of rice (*Oryza sativa*) (**Figures 6, Group 3F**; **7J**) were discovered on one grinding stone and one muller, reflecting the combination of use as a toolset for processing cereals. The individual starch grains of rice are difficult to identify due to their small sizes and lack of unique attributes. However, when they occur in agglomerations, their unique morphological characteristics with a flat surface, tightly grown together, and clearly defined margin between particles confidently could distinguish rice from other plants (Yao, 2016; Li et al., 2019).

## Discussion

Through this micro-botanical study, two modes of subsistence strategy were revealed in ancient Ha Long Bay, corresponding with the different cultural backgrounds in the mainland and offshore areas separately attributed to Ha Long and Trang Kenh cultural assemblages.

A wide range of plant resources was exploited by Bai Ben and Bai Cat Don residents on Cat Ba Island, including taros, yams, acorns, palm, ginger, and beans, as well as foxtail millet, suggesting that the site’s occupants relied heavily on wild plants or horticulture while using limited amounts of rice and millet products. They may have practiced cropping on a small scale or managed exchange activities with neighboring farming groups, as some archaeologists suspected previously (Nguyen, 2009; Nguyen, 2019). The type of plant remains found in Trang Kenh differed from the other two Ha Long cultural sites mentioned above, revealing a food production mode characterized by rice and foxtail millet.

### Indigenous hunter-gatherers and their tropical plants

The underground storage organs (U.S.O.s) from several species of edible aroids (*Colocasia* spp.; *Alocasia* spp.) and yams (*Dioscorea* spp.) were crucial food resources for inhabitants in Ha Long Bay, which could trace back to Cai Beo culture phase about 7000-5000 years BP (Wang et al., 2022). This study confirms that the U.S.O.s remained significant until the late Ha Long phase 3000 years ago. The long-settled hunter-gatherers at Ha Long Bay were highly likely equipped with knowledge of managing and processing U.S.O.s and developed their vegeculture coexisting with cereal agriculture.

The cultural use of aroids and yams extends deep into the local traditions of Southeast Asia and the Pacific regions, and aroids are regarded as fundamental in supporting the development of pre-farming societies (Barker, 2005; Barton and Paz, 2007). Even today, taro (*C. esculenta*) and greater yam (*D. alata*) are cultivated as food crops, while wild yams and *Alocasia* are used as famine foods throughout Southeast Asia (Matthews, 2010; Rugchati and Thanacharoenchanaphas, 2010; Rashmi et al., 2018; Saranraj et al., 2019). Additionally, other kinds of U.S.O.s, such as *Zingiber* sp., traditionally have been exploited or managed by local residents for thousands of years.

The starches from the pith of *Arenga* sp. in Bai Cat Don and Bai Ben indicate the continued exploitation or management of palms by the Neolithic inhabitants on Cat Ba Island. Palms were ubiquitous in southern China and Southeast Asia Neolithic sites that have shown their distinctive phytolith and starch morphologies (Yang et al., 2013; Deng et al., 2020; Zhang et al., 2021). In our previous study, starch grains and phytoliths from Palmae were recovered from Cai Beo (Wang et al., 2022). The representative research at Xincun (ca. 5300 through 4420 cal. years BP) on the Guangdong coast confirmed that sago-type palms (*Caryota* spp.; *Arenga* sp.) were an important food plant before rice in southern China (Yang et al., 2013). However, few species in the genus *Arenga* have been recognized as domesticated from the wild (Pillai et al., 2020). Sugar palm (*Arenga pinnata*) is one of several species of palms culturally crucial to the ethnic groups of Southeast Asia (Sujarwo and Keim, 2021). In Vietnam, sugar palm is grown on the highlands in the central or northern part and utilized for many purposes, especially for making a unique wine from its sap, sometimes called “the Wine of the God” (Dang and Hoang, 2012; Nguyen et al., 2014).

The exploitation of tree nuts, such as *Quercus*, *Canarium*, *and Castanopsis* was common practice throughout Southeast Asia and southern China from late Pleistocene to late Holocene, supported by pollen, macro-botanical findings, and starch grain analysis (Nguyen, 2008; Nguyen, 2014; Yang et al., 2017; Deng et al., 2019; Li, 2020). Rich Fagaceae were identified in Cai Beo (Wang et al., 2022). However, only two starches from *Quercus* were recovered in Bai Cat Don, indicating the possible reduced exploitation of acorns in Ha Long Bay after 4500-4000 years BP.

Acorn is a time-consuming, high-cost, and low-return economic resource that would have entered into the diet only when people are forced to expand their diet breadth (Tushingham and Bettinger, 2013). Therefore, the utilization of acorns may have lost prominence when more edible cereal crops were added to hunter-gatherer diets during the early Ha Long cultural phase around 4500 years BP, as observed in this study. The same shift is reported in the Pearl River Delta of coastal southern China, where acorns were consumed in large quantities from 6000 through 4500 years BP, but then decreased after the arrival of rice agriculture (Li, 2020). Moreover, the remarkable decline of arboreal pollen and increases in Poaceae pollen were recorded at around 4300 to 3300 cal. years BP in the Pearl River Delta (Ma et al., 2020).

### Farmers brought the new plants and new recipes

**Figure 8:**
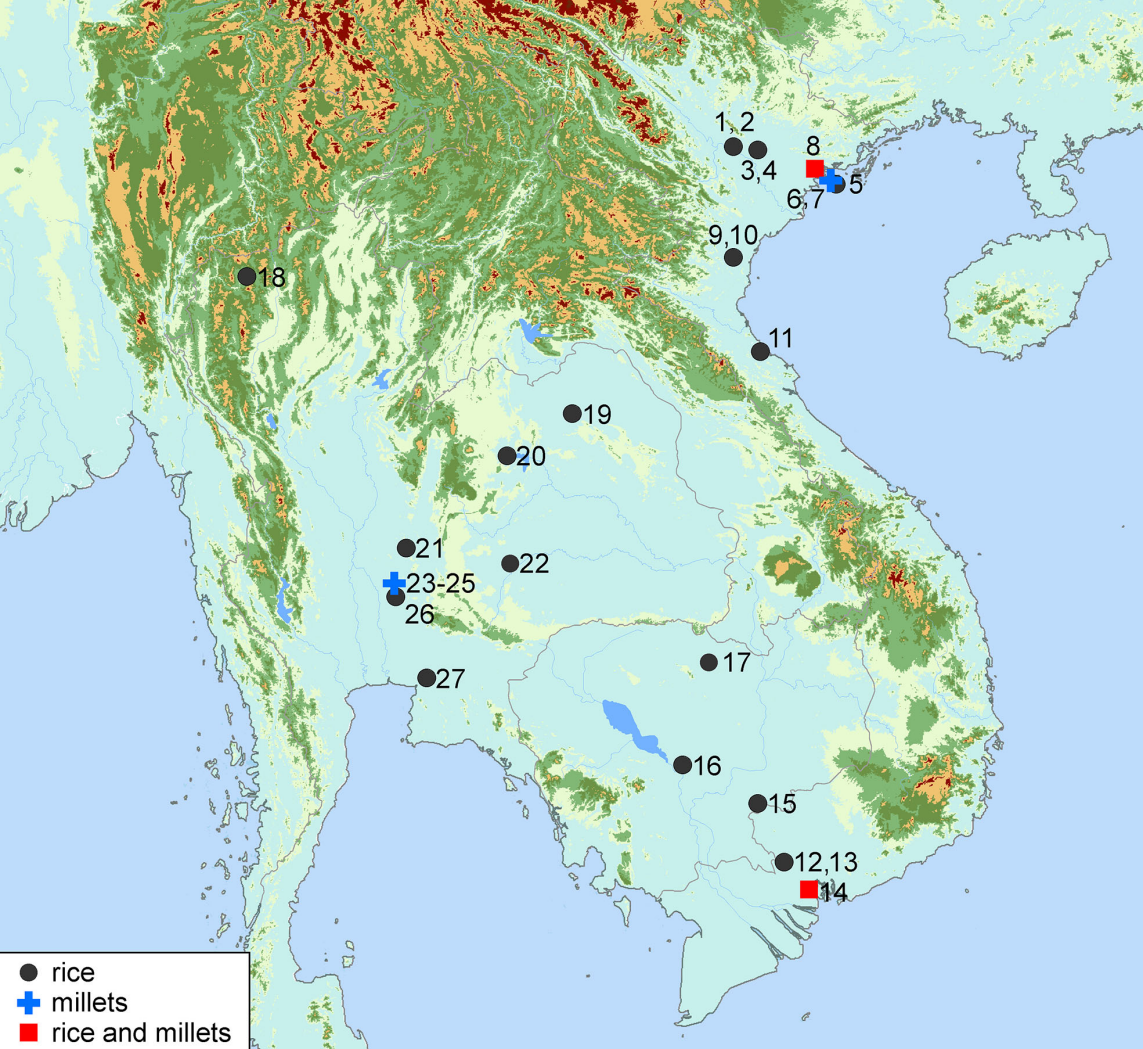
Major sites with the evidence of rice and millets in Mainland Southeast Asia during the Neolithic period. 1. Dong Dau, 2. Thanh Den, 3. Xuan Kieu, 4. Tu Son, 5, Trang Kenh, 6. Bai Ben, 7. Bai Cat Don, 8. Cai Beo, 9. Bai Cu, 10. Bai Man, 11. Thach Lac, 12. An Son, 13. Loc Giang, 14. Rach Nui, 15. Krek 52/62, 16. Samrong Sen, 17. Mlu Prei, 18. Banyan Valley cave, 19. Ban Chiang, 20. Non Nok Tha, 21. Khok Charoen, 22. Ban Lum Khao, 23-25. Non Pa Wai, Non Mak La, Ni Kham Haeng (labeled as millet sites, because no rice was found in the Neolithic period of these three sites), 26. Tha Kae, 27. Khok Phanom Di (see details in **Table 1**).

Millets usually have been absent in the early farming sites in Mainland Southeast Asia (**Figure 8**). In the past decade, the ancient starch grain analysis based on detailed comparative morphological studies on modern millets and their wild relatives has been applied effectively to reconstruct the origins and domestication process of millets in northern China (Yang et al., 2012). A high ubiquity of starch granules from foxtail millets in studied sites showed the superiority of ancient starch grain analysis to recover millet agriculture. This study captures the evidence of foxtail millet in northern Vietnam, revealing an early introduction of foxtail millet highly likely accompanied by rice.

Foxtail millet (*S. italica*) has been found at all three study sites, and Trang Kenh has yielded both foxtail millet and rice, showing the earliest multi-cropping in Mainland Southeast Asia. During this time, with the sea level falling near the present level around 4000 years BP, flatlands with fresh water supply appeared in the upper streams of the coastal basins in northern Vietnam (Ma et al., 2020), providing suitable natural conditions for initial irrigation and agricultural production. As a result, human settlement emerged along the natural levees of the Red River Delta (Funabiki et al., 2012), and the Phung Nguyen group began to develop into a flourishing farming society.

No clear evidence of broomcorn millet has yet been found in northeastern Vietnam; a similar multi-cropping pattern and lacking broomcorn millet have been reported on the Guangdong coast. Broomcorn millet has been discovered more frequently in southwest China and central Thailand (Weber et al., 2010; Dal Martello, 2022) (**Figure 8**). Apparently one problem was due to the limited archaeobotanical work undertaken in northern Vietnam, also noting the limited numbers of samples analyzed in this study. Additionally, according to a modern experiment on the carbonized process of millets, broomcorn millet shows a lower probability of carbonization than seen in foxtail millet in archaeological contexts as its carbonization temperature range is smaller than foxtail millet (Wang and Lu, 2020). Currently, we cannot totally exclude the possibility that the Neolithic farmers in northern Vietnam once may have cultivated a certain amount of broomcorn millet, yet this issue needs further research.

As mentioned, individual starch grains from rice are small and not easy to identify, but the confidence is much higher when they are found in sheets or clusters. The starch grains from rice were documented successfully in many sites dating from 9000 to 7000 cal. years BP in China’s Huai River and Yangtze River region (Yang and Jiang, 2010; Yao et al., 2016; Li et al., 2019; Yang et al., 2021). The numbers of rice starches acquired from Trang Kenh were less than foxtail millet, but this finding does not necessarily indicate that foxtail millet was more important than rice for the ancient Trang Kenh villagers. A dry-grinding simulation experimental study (Li et al., 2020) showed that only a few rice starch grains tend to survive, but foxtail millet could be preserved better under the same dry-grinding conditions. The different preservation bias carbonized cereals may lead to the overrepresentation of rice in Mainland Southeast Asia; carbonized rice seems to be preserved better than carbonized millet (Castillo, 2019). Further study is needed in collaboration with diverse archaeobotanical methods to refine interpretations of these findings.

The presence of starch grains from *Vigna* sp. at Bai Cat Don suggests the region-wide behavior of exploiting beans in Neolithic southern China and Mainland Southeast Asia. For instance, rice bean (*Vigna umbellata*) and azuki bean (*Vigna angularis*) are locally essential legumes that have been domesticated in Southeast and East Asia (Isemura et al., 2011). The macro-remains of *Vigna* spp. were recorded from Baiyangcun and Baodun in southwest China, dating to 5000-4000 cal. years BP (Guedes et al., 2013; Dal Martello et al., 2018), and starches from Kuahuqiao, Xiaohuangshan in the lower Yangtze River basin were dated to 9000-7000 cal. years BP (Yang and Jiang, 2010; Yao et al., 2016). In northern Vietnam, before this study, beans were reported in the Phung Nguyen cultural layer of the Dong Dau site (Nguyen, 2013).

Although the bean starch from Bai Cat Don is too little to determine species, two possibilities are proposed with the newest genetic and archaeobotanical study. First, they were collected locally, noting that the wild progenitors of *Vigna umbellata* have been found in the most extraordinary genetic diversity in Mainland Southeast Asia (Isemura et al., 2011; Castillo et al., 2016). The other possibility is that they may have arrived in northern Vietnam with the spread of agriculturalists, noting that a genetic study discovered azuki bean in mountainous north Vietnam has been most closely related to specimens in the middle Yangtze River basin and had been cultivated in isolation for a long time (Xu et al., 2008; Leipe et al., 2022).

### Spatial-tempo transition of subsistence patterns along with cultural transformation

**Figure 9:**
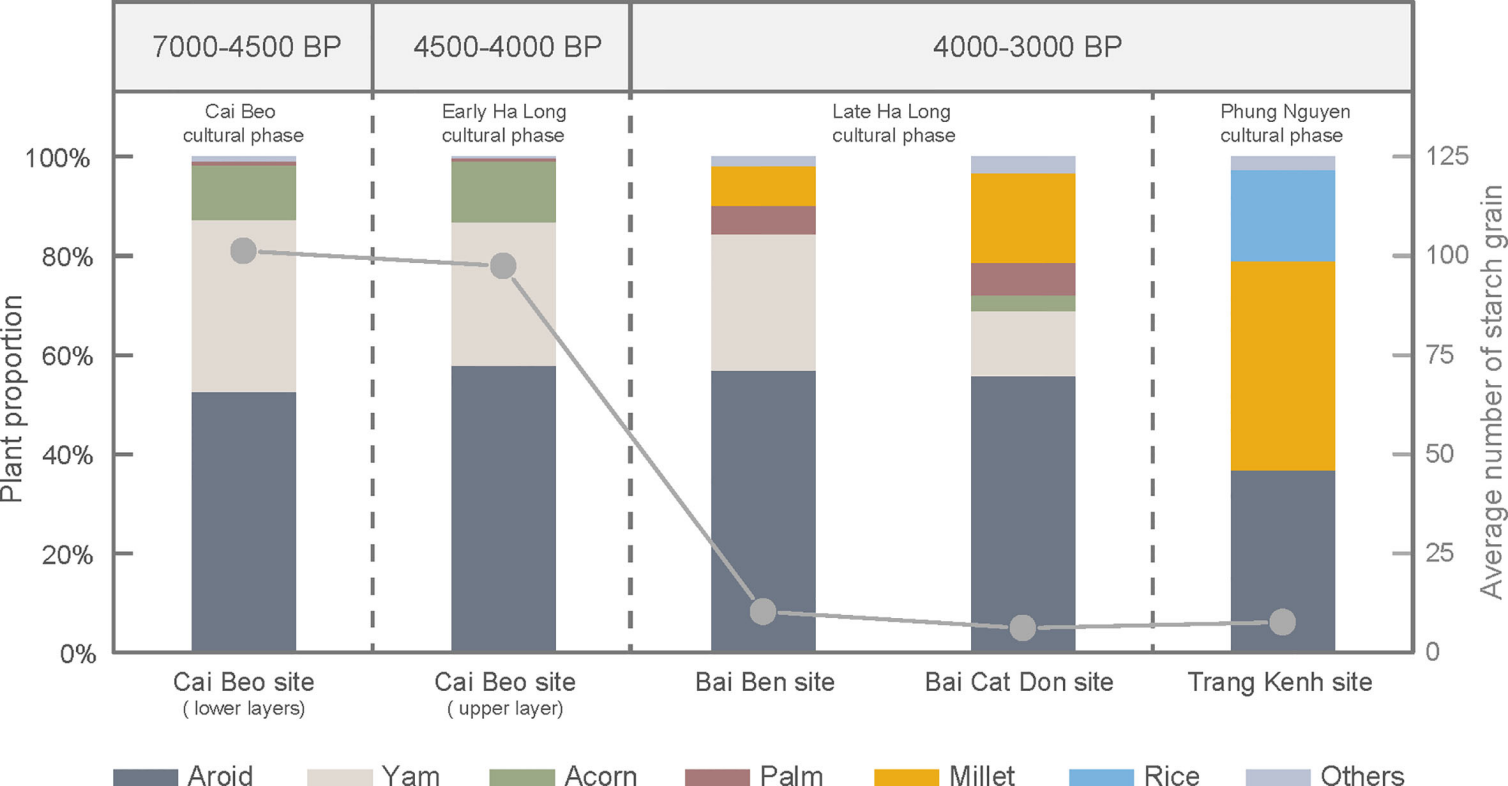
The proportions of different plants recovered from the four studied sites (multicolor histogram correlate to the left coordinate axis), and the average number of starch grains recovered (gray dots and solid line correlate to the right coordinate axis). The average number of starch grains found on each stone tool from the Cai Beo and Early Ha Long phases was 100, but the number dropped in the Late Ha Long and Phung Nguyen phases to below 10 grains on each tool. Clearly, the numbers of rice and millet increased through time.

The plant consumption history from 7000 through 3000 years BP in Ha Long Bay could be reconstructed by integrating our previous findings (Wang et al., 2022) and the new information from this study. For the earlier stage, no dramatic difference was noticed in the use of plant species and quantities from the Cai Beo cultural phase (ca. 7000-4500 cal. years BP) to the Early Ha Long cultural phase (ca. 4500-4000 cal. years BP), indicating the cultural continuity of plant utilization mode; the Cai Beo residents on the Cat Bat Island relied heavily on aroids, yams, and acorns (**Figure 9**). However, as early as 4500-4000 cal. years BP, the appearance of rice phytoliths in the Ha Long phase at the Cai Beo site, implies the first farming expansion into Ha Long Bay (Wang et al., 2022). Furthermore, ancient DNA analysis of the Hon Hai Co Tien human remains of the Ha Long phase has indicated an initial gene admixture of East Asians with the Hoabinhian hunter-gatherers at 4381-3926 cal. years BP (McColl et al., 2018).

As presented in this study, the transition of subsistence strategies became more apparent in the Late Ha Long culture around 4000 years BP. The average starch grains recovered on stone tools from Bai Ben and Bai Cat Don experienced a sharp decrease compared with the preceding phases in the Cai Beo site (**Figure 9**). The number of grinding stone tools from this period gradually declined, representing another cultural indicator of a possible subsistence transition. Although the diversity of utilized plant species became less (Wang et al., 2022), the persistent group (the late Ha Long group) on Cat Ba Island continued to exploit the same plant resources, particularly involving extensive consumption of aroids and yams. The diverse ecosystems and landscapes on Cat Ba Island support high species biodiversity (Van et al., 2010), providing the advantage of rich territorial animal and plant resources from inland areas, while continuing to accommodate the ancient Ha Long people living on their traditional lifestyles of mixed hunting, fishing, and gathering economies (Nguyen, 2019; Nguyen, 2020).

In a broader context in prehistoric southern China and Southeast Asia, hunting and gathering often continued as essential subsistence activities even after the arrival of animal and plant domesticates (Castillo et al., 2018; Li, 2020; Deng et al., 2022a), and the expansion of early farmers did not lead to the total extinction of the long-established hunter-gatherers in Southeast Asia (Higham et al., 2011). For example, even after several hundred years of the introduction of agriculture in the Mekong Delta region, hunting and foraging remained dominant at Rach Nui (Castillo et al., 2018), and this pattern contrasted in cultural materials with contemporary highly developed agriculture communities.

Given that the majority of Ha Long cultural inhabitants were descendants of indigenous hunter-gatherers and exhibited distinct cultural contexts with other farming groups in northern Vietnam, the foxtail millets discovered in Bai Ben and Bai Cat Don were perhaps acquired through exchanges or trades. As mentioned, from an archaeological perspective, the Ha Long group appears to have sustained contacts and exchanges with other groups, such as Phung Nguyen, Ha Giang, Mai Pha (Lang Son), Hoa Loc, and offshore islands along the South China Sea coast (Ha, 2005; Nguyen, 2005b; Nguyen, 2019). However, another possibility is that the people of Bai Ben and Bai Cat Don practiced a specific scale of millet farming on their own, as a stone knife-like fragment, typically used for crop harvesting, has been found at Ba Vung (Chen, 2007), one of the Ha Long cultural sites. This possibility can be explored through further research.

At Trang Kenh, which represented a typical Neolithic farming site with extensively worked jade remains, the discovered plant resources showed that people relied on cereal-based farming (**Figure 9**). This subsistence pattern coincided with a cultural background of agricultural communities that depended heavily on cultivated crops, which contrasts with hunter-gatherers that explored a broad range of wild plant resources. The limited discovered amount of rice and foxtail millet from Trang Kenh may have been due to stone artifacts from this site mainly related to nephrite ornaments manufacturing. The stone tools associated with daily life were far fewer. More findings undoubtedly will be recovered in the residential areas in future work.

## Conclusion

The archaeobotanical studies from three Neolithic sites in Ha Long Bay reveal that rice and millets already existed in northern Vietnam around 4000 years BP. Based on the comparison of cultural assemblage and chronology in the study and neighboring regions, the multi-cropping likely can be traced back to 4500- 4000 years BP in the northern part of Mainland Southeast Asia. The coastline along the South China Sea probably contributed a role in expediting the early cultural interaction and rapid movement of farming groups into northern Vietnam. However, the cultural contacts with southwest China through inland routes are apparent as well, which could be exemplified by the discoveries of *Yazhang* jade blades and other identical pottery and lithic tools of southwest China characteristics from the Phung Nguyen cultural sites (Hoa, 1996). Thus, the cultural landscapes of northern Vietnam at 4000 years BP are diverse and complex.

Based on this study, we can reconstruct two plant-based subsistence patterns, which coexisted in northeastern Vietnam from 4000 through 3000 years ago, and two different cultural groups practiced them, perhaps with their own cultural and biological backgrounds (Lipson et al., 2018; McColl et al., 2018; Matsumura et al., 2019; Matsumura et al., 2021). Nevertheless, many questions remain unclear. Further research is needed in collaboration with a refined chronology, and macro-plant remains, in order to disclose more fully comprehensive information about plant-based subsistence strategies correlated with early Neolithic settlements in northern Vietnam.

## Data availability statement

The original contributions presented in the study are included in the article/supplementary material. Further inquiries can be directed to the corresponding author.

## Author contributions

H-CH, WW, and KN conceived and designed the study. KN and HL provided the archaeological samples. H-CH, WW, KN, HL, and CZ collected the study samples. WW completed sample identification and data analysis. XY assisted with the starch identification. WW and H-CH wrote the manuscript. MTC edited the manuscript. All authors contributed to the article and approved the submitted version.

## Funding

This study was funded in part by the Australian Research Council (Grant Number: DP190101839), the National Natural Science Foundation of China (Grant No. 41930323), and the Chinese Scholarship Council (Grant Number: E94962376).

## Acknowledgments

We appreciate Director Dr. Doi Nguyen (Institute of Archaeology, Vietnam Academy of Social Science, Hanoi) and Deputy Director Mr. Do Xuan Trung (Hai Phong Museum) for their support of this study.

## Conflict of interest

The authors declare that the research was conducted in the absence of any commercial or financial relationships that could be construed as a potential conflict of interest.

## Publisher’s note

All claims expressed in this article are solely those of the authors and do not necessarily represent those of their affiliated organizations, or those of the publisher, the editors and the reviewers. Any product that may be evaluated in this article, or claim that may be made by its manufacturer, is not guaranteed or endorsed by the publisher.
